# Laminoplasty and Laminectomy Hybrid Decompression for the Treatment of Cervical Spondylotic Myelopathy with Hypertrophic Ligamentum Flavum: A Retrospective Study

**DOI:** 10.1371/journal.pone.0095482

**Published:** 2014-04-16

**Authors:** Huairong Ding, Yuan Xue, Yanming Tang, Dong He, Zhiyang Li, Ying Zhao, Yaqi Zong, Yi Wang, Pei Wang

**Affiliations:** Department of Orthopedics, Tianjin Medical University General Hospital, Heping District, Tianjin, China; Georgetown University Medical Center, United States of America

## Abstract

**Objective:**

To report the outcomes of a posterior hybrid decompression protocol for the treatment of cervical spondylotic myelopathy (CSM) associated with hypertrophic ligamentum flavum (HLF).

**Background:**

Laminoplasty is widely used in patients with CSM; however, for CSM patients with HLF, traditional laminoplasty does not include resection of a pathological ligamentum flavum.

**Methods:**

This study retrospectively reviewed 116 CSM patients with HLF who underwent hybrid decompression with a minimum of 12 months of follow-up. The procedure consisted of reconstruction of the C4 and C6 laminae using CENTERPIECE plates with spinous process autografts, and resection of the C3, C5, and C7 laminae. Surgical outcomes were assessed using Japanese Orthopedic Association (JOA) score, recovery rate, cervical lordotic angle, cervical range of motion, spinal canal sagittal diameter, bone healing rates on both the hinge and open sides, dural sac expansion at the level of maximum compression, drift-back distance of the spinal cord, and postoperative neck pain assessed by visual analog scale.

**Results:**

No hardware failure or restenosis was noted. Postoperative JOA score improved significantly, with a mean recovery rate of 65.3±15.5%. Mean cervical lordotic angle had decreased 4.9 degrees by 1 year after surgery (P*<*0.05). Preservation of cervical range of motion was satisfactory postoperatively. Bone healing rates 6 months after surgery were 100% on the hinge side and 92.2% on the open side. Satisfactory decompression was demonstrated by a significantly increased sagittal canal diameter and cross-sectional area of the dural sac together with a significant drift-back distance of the spinal cord. The dural sac was also adequately expanded at the time of the final follow-up visit.

**Conclusion:**

Hybrid laminectomy and autograft laminoplasty decompression using Centerpiece plates may facilitate bone healing and produce a comparatively satisfactory prognosis for CSM patients with HLF.

## Introduction

Cervical spondylotic myelopathy (CSM) is a common cause of atraumatic quadriplegia in adults [1]. Surgical decompression is the most common procedure for the treatment of CSM patients with moderate to severe or progressive neurologic deficits [2], [3]. Several techniques with anterior, posterior, or combined approaches have been developed [4]–[6], with posterior decompression being the logical treatment for a spinal cord that has been compressed dorsally by a hypertrophic ligamentum flavum [7]–[9]. Laminoplasty is a potential posterior approach because it preserves posterior bony elements of the spinal canal and reduces the risk of post-laminectomy kyphotic deformity [10], [11]. Compared with holding the door open with a suture tethered to the facet capsule or anchored to the lateral mass, laminoplasty with a Centerpiece plate can provide rigid fixation and reduce the risk for laminae reclosure or sinking from fragility of the fixation [3], [12]–[15].

**Figure 1:**
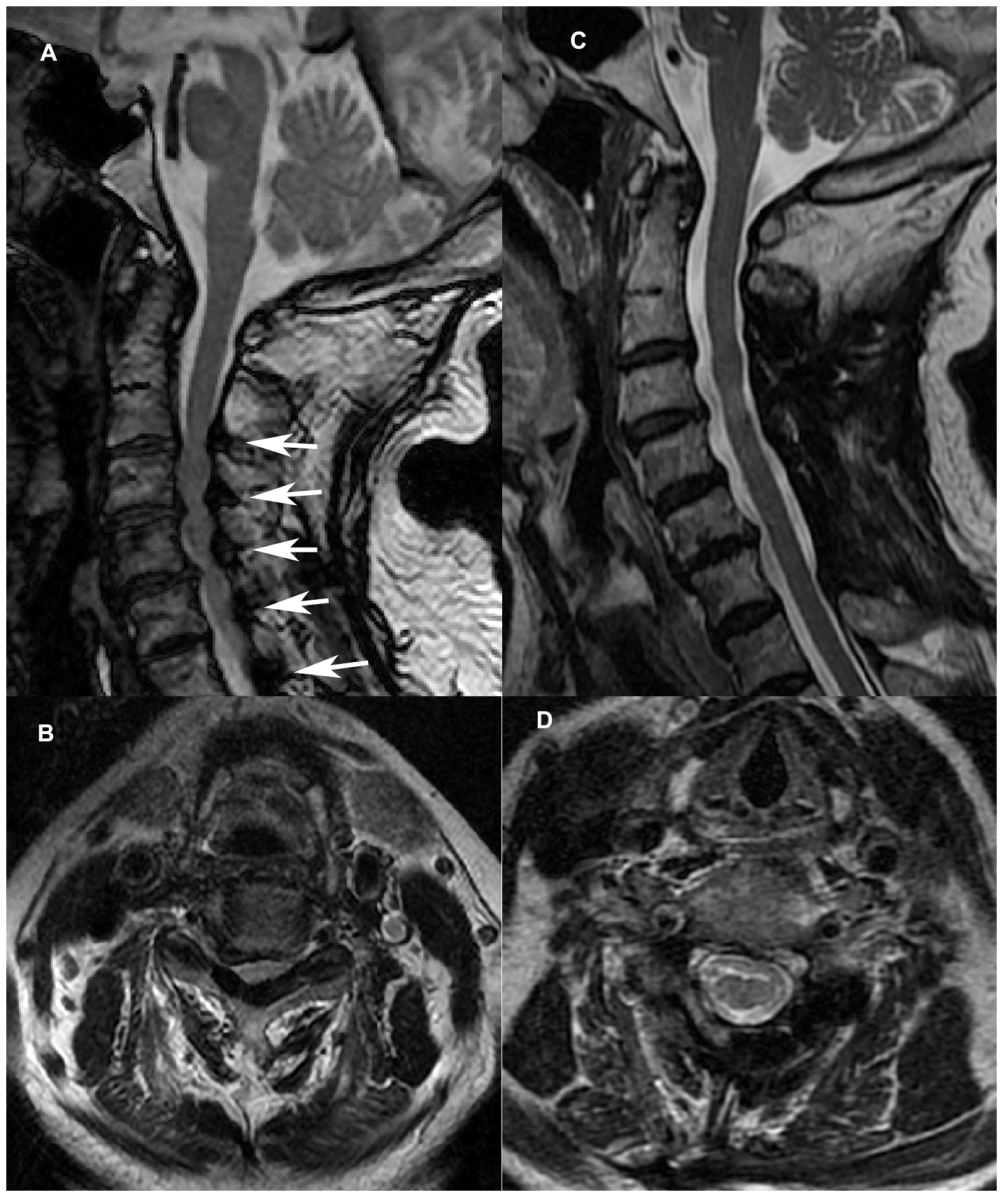
Pre- and postoperative MRI of a 57-year-old man with multilevel cervical canal stenosis. (A) Preoperative sagittal and (B) axial views of this MRI show the cervical spinal cord compressed by degenerative disk herniation at C3–4, C4–5 and C5–6 with five-level hypertrophic ligamentum flavum (arrows). (C) Postoperative sagittal and (D) axial MRI views 1 year after surgery demonstrate satisfactory decompression of the spinal cord. The dural sac has expanded significantly, and no restenosis is observed. MRI, magnetic resonance imaging.

In CSM patients with hypertrophic ligamentum flavum (HLF), the dorsal canal elements are one cause of compression of the spinal cord (Fig. 1A, B). To remove the HLF completely while partially preserving the posterior wall of the spinal canal, we have developed a hybrid decompression protocol, laminectomy at the C3, C5, and C7 levels and laminoplasty at the C4 and C6 levels with spinous process autograft using the Centerpiece Plate Fixation System (Medtronic Sofamor Danek, Memphis, TN, USA). The aim of this retrospective study was to perform a preliminary review of the results of this hybrid decompression protocol.

## Materials and Methods

The study was approved by the Medical Ethics Committee of Tianjin Medical University General Hospital and performed according to the principles of the Declaration of Helsinki. Written informed consent was obtained from the patient whose images were used in this article, but the requirement for informed consent of the other patients was waived by the ethics committee because of the retrospective nature of this research.

### The Centerpiece Plate Fixation System

**Figure 2:**
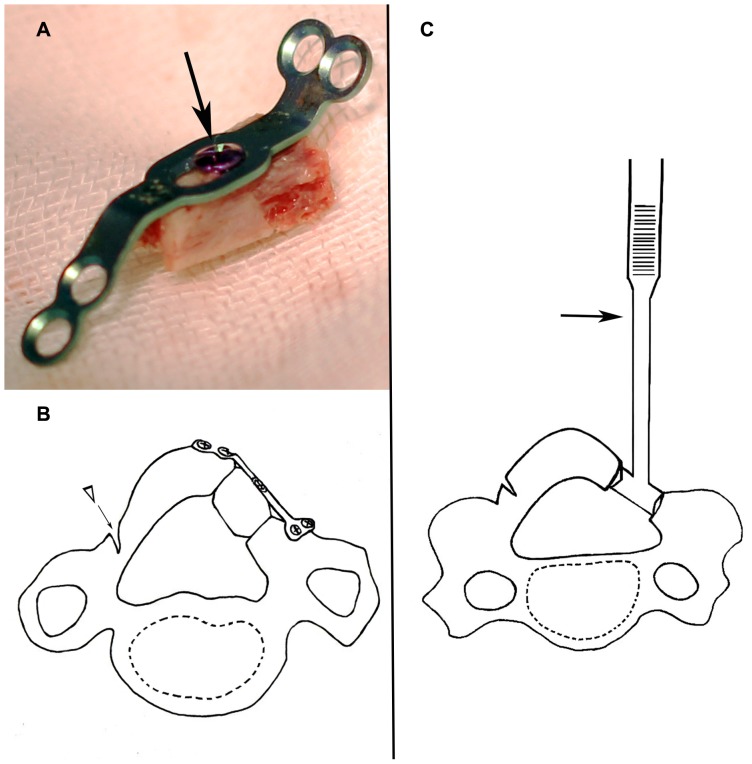
Reconstruction of elevated lamina. (A) Autologous spinous process/graft plate construct. The spinous process is attached to the graft plate and secured by one mini-screw (arrow) through a pre-drilled center hole in the spinous process. (B) Fixation of elevated lamina. On the open side, the elevated lamina is fixed by the autologous spinous process/graft plate construct and mini-screws; on the hinge side, bone chips harvested from resected laminae are inserted into the residual gutter to facilitate bone union. (C) Measurement of elevated lamina. The height of lamina elevation is measured by a bone trial (arrow), which can aid in the selection of the appropriate Centerpiece plate and bone block.

The Centerpiece Plate Fixation System (Medtronic Sofamor Danek, Memphis, TN, USA) has been approved by the United States Food and Drug Administration (Regulation Number: 21 CFR 888.3050). The Centerpiece system contains open-door plates and graft plates. Open-door plates are commonly used in classic cervical laminoplasty procedures [12], [14], [15]; in our procedure, we used the graft plates and spinous process autograft (Fig. 2A) to achieve rigid fixation and bone healing. Open-door plates are available in sizes from 8 to 18 mm in 2-mm increments. An oval-shaped center screw hole in the graft plate allows for fine adjustments of the plate on the graft (Fig. 2A).

### Study Participants

During a consecutive 3-year period from February 2009 through April 2012, 283 patients underwent hybrid posterior decompression in our hospital. The current study retrospectively reviewed 116 CSM patients with HLF who underwent this procedure. The researchers selected patients with CSM caused only by HLF (dorsal compression of the spinal cord owing to HLF) and patients with CSM caused by the combination of anterior disease plus HLF (dorsal compression of the spinal cord owing to HLF and anterior disease such as ossification of the posterior longitudinal ligament [OPLL], degenerative disk disease [DDD], and congenital pathologies). The inclusion criteria were: 1) a clear diagnosis made by history, physical examination, plain radiographs, computed tomography (CT), and magnetic resonance imaging (MRI); 2) complete pre- and postoperative imaging and clinical data records; 3) a minimum of 12 months’ postoperative follow-up. Exclusion criteria were: 1) CSM patients with multilevel anterior compression only; e.g., consecutive OPLL or DDD, but without HLF; and 2) patients lost to follow-up. Of the 116 patients (89 men, 27 women [mean age 61.9 years], 55 also had segmental or localized OPLL, 41 also had cervical degenerative disk herniation (DDH), 11 also had developmental spinal canal stenosis (DSCS), and 9 had ossification of ligament flavum (OLF). Thirteen of 116 patients had single-level spinal canal stenosis, 29 had two-level stenosis, 48 had three-level stenosis, and the remaining 26 patients had four- or five-level stenosis.

### Operative Technique

Local anesthesia was provided for 75 patients, and 41 patients underwent general anesthesia and endotracheal intubation. The type of anesthesia were chosen by the anesthesiologist. Patients were placed in lateral decubitus position with the neck slightly flexed.

### Surgical Procedure (Video S1)

**Figure 3:**
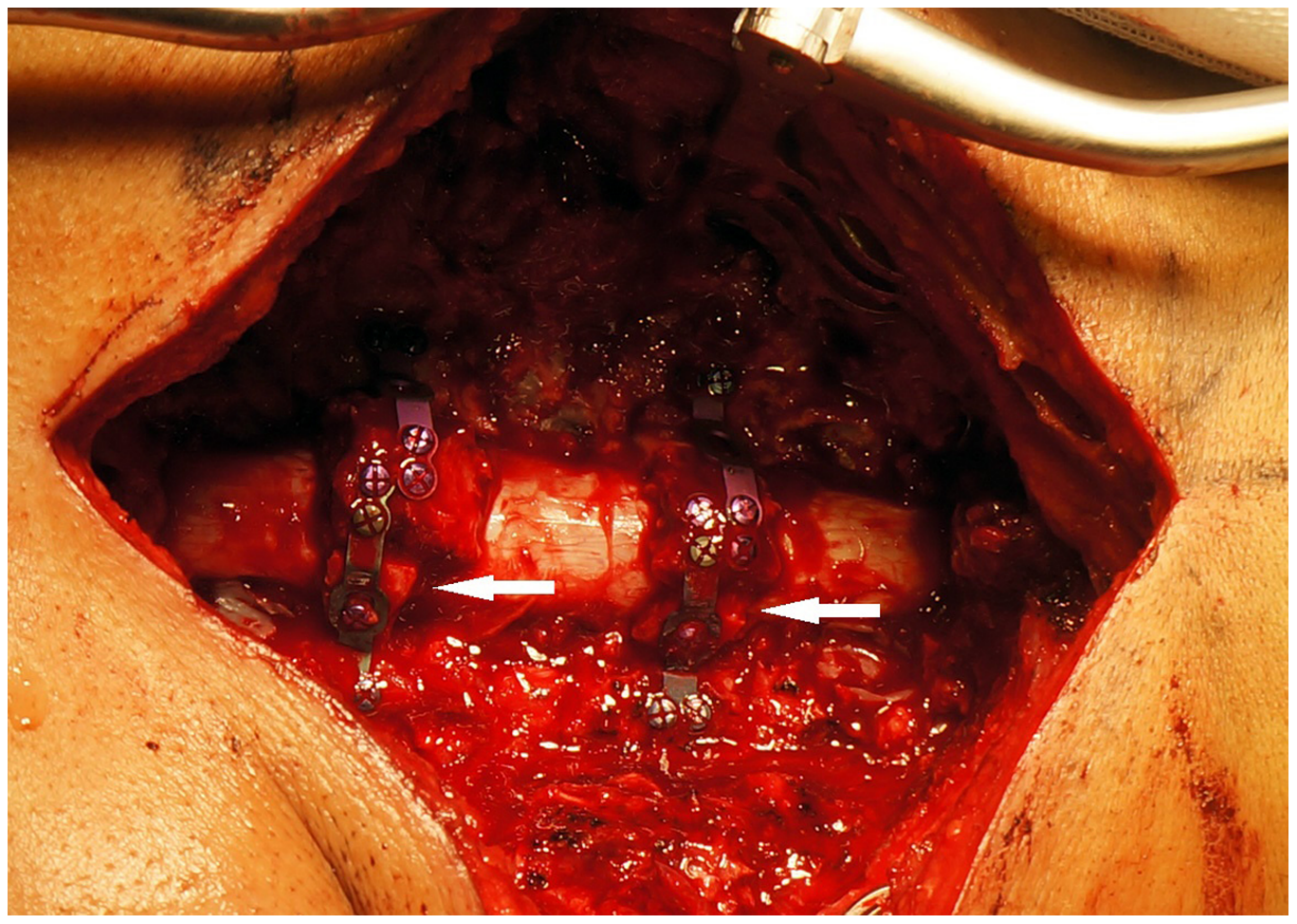
Intraoperative photograph of the hybrid decompression protocol. Between C3 and C7, the C4 and C6 laminae are reconstructed and the C3, C5, and C7 laminae and ligamentum flavum are removed. The opened laminae are fixed with Centerpiece plates with shaped autologous spinous process (arrows). Four Centerpiece plates were used in this patient.

**Figure 4:**
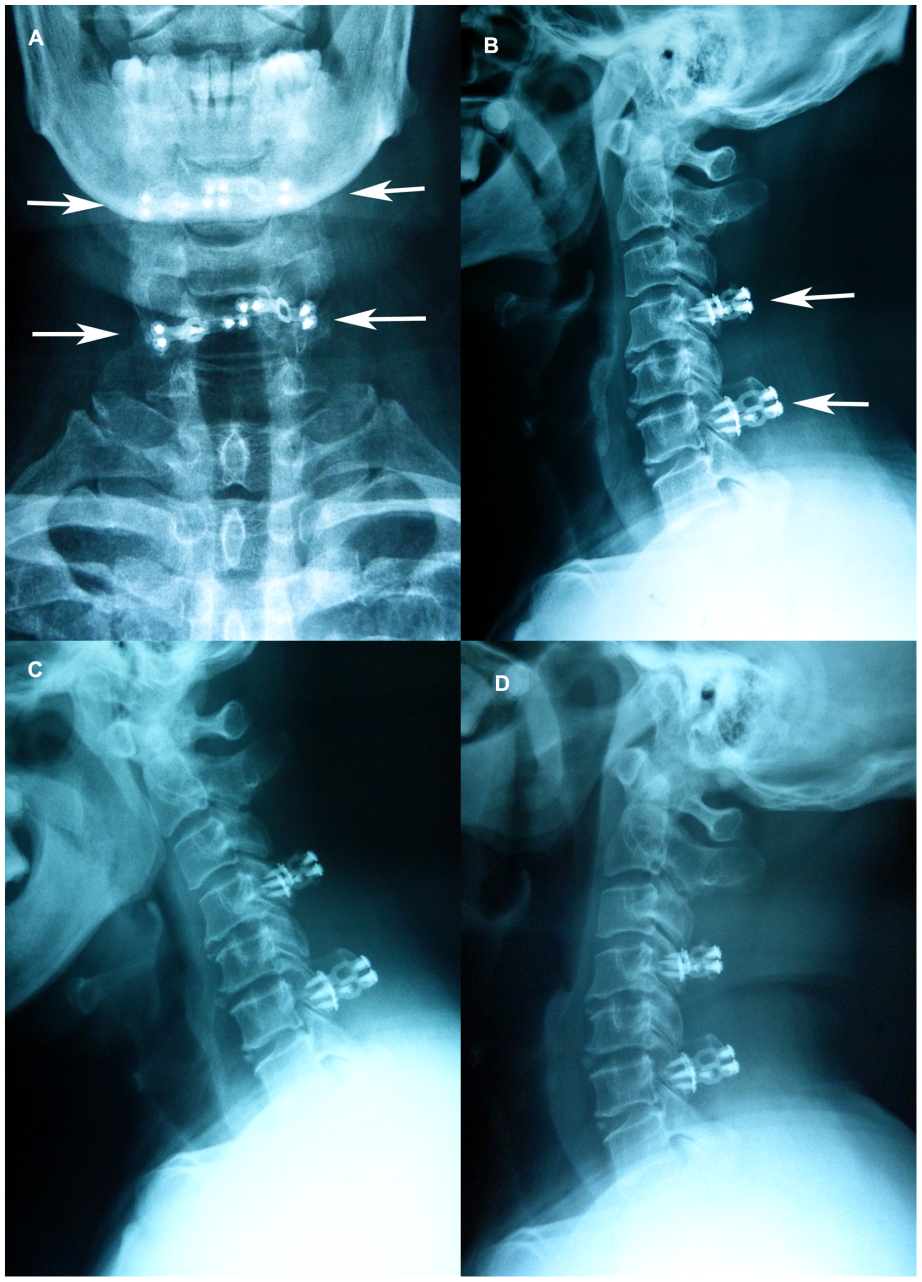
One-year-postoperative radiographs. (A) Anteroposterior and (B) lateral postoperative radiographs show the C3, C5, and C7 laminectomies and C4 and C6 laminoplasties with spinous process autograft Centerpiece plate fixation. (C), (D): Postoperative maximal flexion and extension lateral radiographs. No kyphosis or hardware failure is observed.

**Figure 5:**
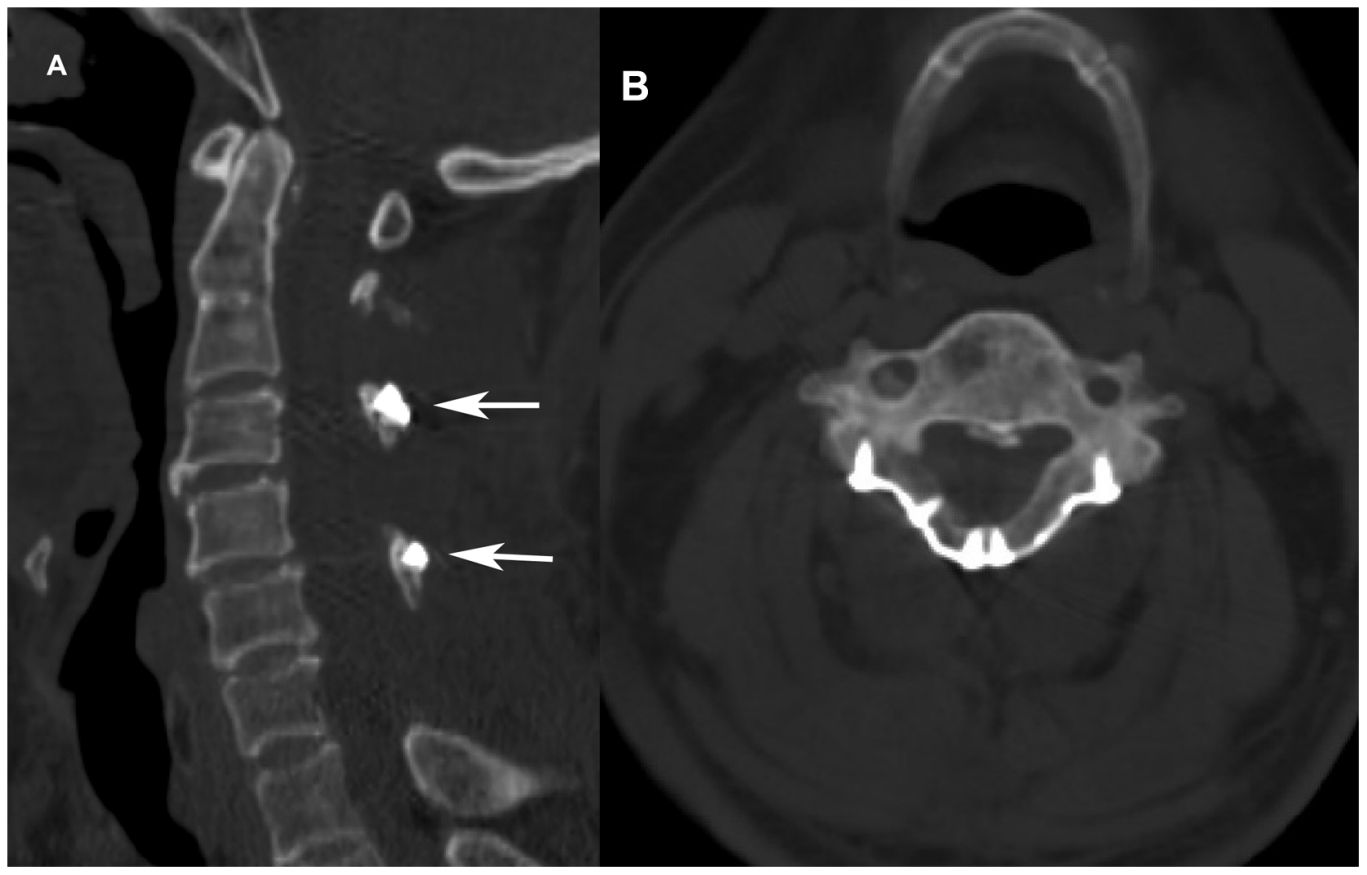
Postoperative CT scan of a patient 6 months after surgery. (A) Sagittal CT scan shows enlargement of the cervical spinal canal after posterior hybrid decompression. (B) Axial CT scan shows the reconstruction of a continuous and stable bony laminar arch. The hinge has completely healed with cortical bone on both its dorsal and ventral surfaces; the junction of bone block and host bone on the open side are bridged by cancellous bone.

A standard posterior approach was employed, with a midline incision created to expose the cervical laminae from the caudal edge of C2 to the cranial edge of T1 and extended laterally for complete exposure of the dorsal cortex of the bilateral facet joints. Each spinous process was removed and shaped for grafting. After removal of the C3, C5, and C7 laminae and the ligamentum flavum in the area of decompression, the C4 and C6 laminae were reconstructed. Gutters were created on both sides of the laminae by completely removing the dorsal cortex and thinning the ventral cortex at the margin between the lamina and the lateral mass using a 4.0-mm burr. A 1.0-mm Kerrison rongeur was used to complete the ventral cortex cut on the open (right) side of the lamina. The laminae were then pulled posteriorly and laterally until the dural sac was thoroughly expanded and typical dural pulsation could be seen. During elevation of the laminae, fibrous adhesions between the dura and the ventral surface of the laminae were divided using nerve dissectors. Epidural venous plexus hemorrhage was controlled with bipolar electrocautery or application of Gelfoam (Pfizer, Inc., New York, NY, USA). An autologous spinous process/graft plate construct (Fig. 2A) was used to hold the door open (Fig. 2B), and appropriate lengths of Centerpiece plate and bone block were determined by the height of the lamina elevation, which could be measured using bone trials (Fig. 2C). On the hinge side, bone chips harvested from the resected laminae were inserted into the residual gutter (Fig. 2B), and another graft plate was applied for fixation. All the Centerpiece plates were securely fixed using 5- or 7-mm mini screws from the plate set. Finally, the residual hypertrophic ligamentum flavum and adjacent laminae extending under the edge of the reconstructed laminae were resected completely using a Kerrison rongeur. (Figs. 3, 4, 5).

### Postoperative Care

Patients were required to stay in bed for the first week after surgery. They were then allowed to stand and walk with a cervical collar. The collar was recommended to be worn for 4 weeks postoperatively, after which patients were encouraged to perform gradual mobilization in flexion-extension, rotation, and side bending as tolerated.

### Evaluation of Surgical Outcome

Surgical outcomes were assessed by review of clinical data and imaging examinations from before surgery; 3, 6, and 12 months postoperatively; and annually thereafter.

### Clinical Evaluation

Neurologic conditions were assessed using the scoring system developed by the Japanese Orthopaedic Association (JOA), and functional improvements in JOA scores were expressed as recovery rate (RR) [16]. The RR was determined as follows: RR (%) = (postoperative JOA score – preoperative JOA score)/(17 [normal functional score] – preoperative JOA score)×100. The RR for each patient was classified into 4 grades: those greater than 75% were categorized as excellent for activities of daily living, those that were 50%–74% were categorized as good, those that were 25%–49% were categorized as fair, and those that were less than 24% were categorized as poor. Postoperative neck pain was measured using a 10-point (10-cm) visual analog scale (VAS).

### Radiologic Evaluation

**Figure 6:**
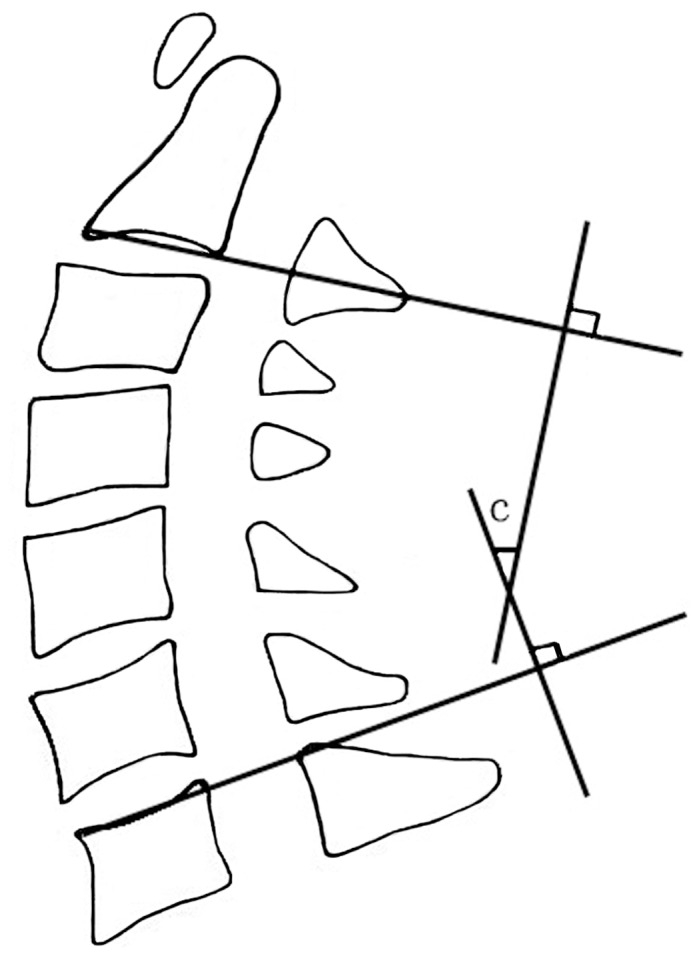
Cobb’s method for measuring cervical lordosis. The cervical lordotic angle is measured according to Cobb’s method on a lateral neutral radiograph: the angle (c) is formed by the two lines perpendicular to the two lines parallel to the inferior endplates of the C2 and C7 vertebral bodies. When the C7 vertebra is not well visualized on lateral radiographs, the inferior plate of C6 is used.

**Figure 7:**
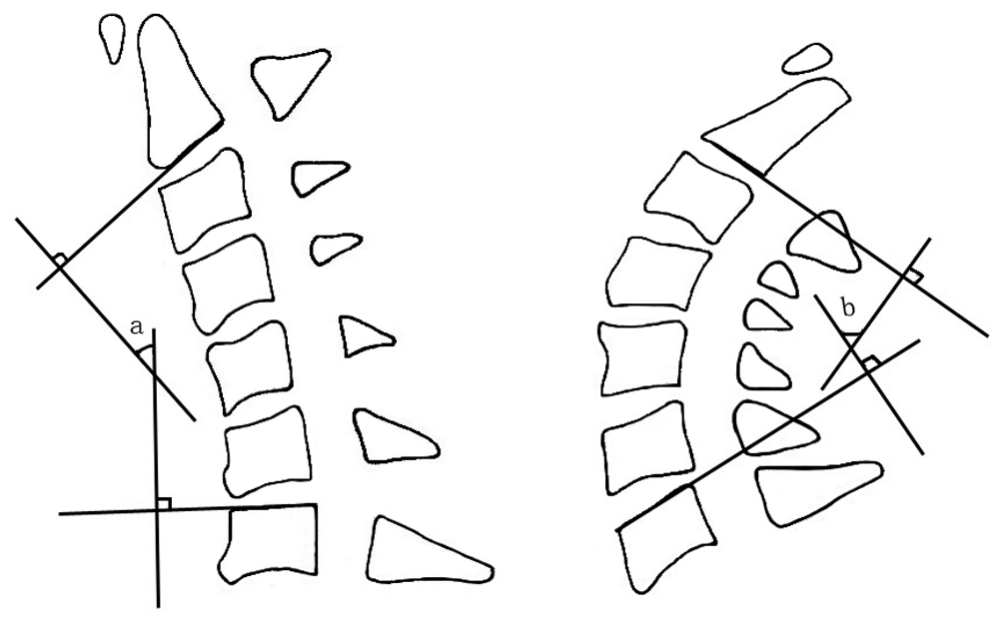
Range of motion of the cervical spine. Angles a and b are created by a line parallel to the inferior aspect of the C2 vertebral body and a line parallel to that of the C7 vertebral body and were measured on maximal flexion and extension lateral radiographs. Total ROM value was obtained by adding these angles (ROM = angle a+angle b).

Alignment of the cervical spine was assessed using the lordotic angle measured between C2 and C7 according to Cobb’s method on a neutral-position lateral plain radiograph (Fig. 6). Cervical range of motion (ROM) was measured as the difference between Cobb angles at maximal flexion and extension on anteroposterior (AP) radiographs (Fig. 7).

The AP diameter of the spinal canal at the reconstructed level was measured as the distance between the posterior surface of the vertebral body and the innermost cortical surface of the reconstructed lamina at the C4 or C6 level, based on a cross-sectional view on CT scan. Bone healing was defined as the presence of cancellous or cortical bridging bone, or the absence of any radiolucent defect on the hinge or open side, on each reconstructed lamina on CT scan.

Dural sac expansion was evaluated by measuring the cross-sectional area of the dural sac at the maximally compressed level on T2-weighted magnetic resonance images. The sagittal sectional area of the dural sac was measured using a cursor to trace the outline of the spinal cord on the maximally compressed slice, and the cross-sectional area was calculated by integrating the sagittal sectional area [17]. Surface-rendering software (GE Healthcare, Waukesha, WI, USA) was used for all measurements.

To estimate the posterior shift of the spinal cord, the distance from the rigid anterior wall of the spinal canal to the posterior edge of the spinal cord at each vertebral body or disk level between C2 and C7 was measured on MRI [18].

Operative duration, estimated blood loss, and intra- and postoperative complications (such as C5 palsy, axial pain, dural tear, cerebrospinal fluid leakage, infection, kyphosis, hardware failure, and lamina reclosure) were also noted.

### Statistical Analysis

Results were analyzed using PASW Statistics for Windows, Version 18.0 (SPSS Inc., Chicago, IL, USA). Descriptive statistics were used for demographics, and Student’s t-test was used for continuous variables. Significance was set at P<0.05.

## Results

Patients’ demographic and primary clinical data are presented in Table 1.

**Table 1:** Demographic and primary clinical data of the patients.

patients	Mean± SD	Range	p
Age (year)	61.9±10.6	41–82	
Sex (male/female)	89/27		
Duration of symptoms (months)	26.2±31.8	3–120	
Levels of surgery	C3–C7 (n = 116)		
Operation time (min)	142.2±28.5	100–210	
Blood loss (ml)	159.9±66.0	100–300	
Follow-up period (months)	28.4±9.7	12–50	
Pre−/postoperative JOA score	(7.0±3.2)/(13.4±2.1)	(1–12)/(9–17)	0.000
JOA Recovery rate (%)	65.3±15.5	33.3–100	
Pre−/postoperative spinal SD^a^ (mm)	(9.6±2.4)/(16.3±1.9)	(6–15)/(12–21)	0.000
Pre−/postoperative Cervical ROM^b^ (degree)	(36.8±13.5)/(33.6±9.1)	(19–63)/(17–59)	0. 252
Pre−/postoperative Cob angle^c^ (degree)	(13.4±8.2)/(8.4±12.6)	(5–29)/(−12–37)	0.004
Postoperative Axial pain (n = 4)	2.3±0.65 (3.5%)		

a SD: sagittal diameter, ^b^ROM: range of motion, ^c^Cob angle: Cob angle measured at C2–C7.

### Clinical Outcome

The mean postoperative JOA score improved significantly (P<0.001) compared with preoperative JOA. One hundred one patients (87.1%) were found to have either excellent or good recovery rate. Mean JOA recovery rate was 65.3%. No patient’s condition worsened after surgery.

### Radiologic Outcome

Mean cervical lordotic angle decreased from13.3 degrees preoperatively to 8.4 degrees by 1 year after surgery (P = 0.004), but neurologic function did not deteriorate. Postoperative cervical ROM was preserved, and the difference between postoperative and preoperative cervical ROM was not significant (P = 0. 252).

CT scans showed a mean 6.7-mm increase in sagittal diameter at the reconstructed levels after surgery. The mean bone healing rates 3 months after surgery were 94.4% on the hinged side and 82.3% on the open side (Fig. 5B), and 1 year after surgery, evidence of bone healing was seen in all patients (Table 2).

**Table 2:** Mean bone healing rates on the hinge and open side after surgery (%).

n = 116	3 months		6 months		1 year	
	Hinge side (n)	Open side (n)	Hinge side (n)	Open side (n)	Hinge side (n)	Open side (n)
C4	92.2% (107)	84.5% (98)	100% (116)	94.0% (109)	100% (116)	100% (116)
C6	96.6% (112)	80.2% (93)	100% (116)	90.5% (105)	100% (116)	100% (116)
Mean	94.4% (109.5)	82.3% (95.5)	100% (116)	92.2% (107)	100% (116)	100% (116)

Mean dural sac cross-sectional area at the maximally compressed level, as assessed on MRI, significantly increased from 95.1 mm^2^ before surgery to 164.0 mm^2^ 3 months after surgery (P<0.001), with a mean 3.3-mm posterior shift of the spinal cord. MRI data for dural sac cross-sectional area and posterior shift of the spinal cord at final follow-up were similar to those 3 months after surgery, with no significant differences detected (Table 3). MRI data 1 year after surgery also showed satisfactory expansion of the spinal cord, and no significant concave defect on the dorsal side of the dural sac between the reconstructed levels was found in any patient (Fig. 1C, D).

**Table 3:** Postoperative spinal cord drift-back distance (mm) and cross-sectional area of the dural sac (mm^2^) at 3 months and 1 year after surgery (Mean± SD).

	3 months (Mean± SD)	1 year (Mean± SD)	p
Spinal cord drift-back distance (mm)	3.31±0.89	3.28±0.92	0.89
Cross-sectional area of the dural sac^a^ (mm^2^)	183.4±34.6	183.5±36.0	0.99

a Cross-sectional area of the dural sac: Cross-sectional area of the dural sac measured at the maximally compressed level.

### Complications

The dura mater was torn during the operation in six patients. Only one patient presented with leakage of cerebrospinal fluid postoperatively, and this patient recovered after revision of the wound and application of a pressure dressing. No severe intraoperative complications such as spinal cord injury or bleeding occurred. No patient had fever, allergic reaction, or infection, and sinking of the laminae or implant failure, such as screw loosening, displacement, and rupture, was not observed (Figs. 1C, D; 4A, B; 5). No patient was noted to have kyphosis or C5 radiculopathy. Only four patients complained of postoperative axial pain. Mean VAS score was 2.3, and all patients’ pain resolved within 3 months with conservative treatment. No patient reported severe neck pain that interfered with activities of daily living.

## Discussion

The pathophysiology of CSM with HLF includes direct dorsal compression (Fig. 1A, B). During neck extension, an HLF buckles and impinges on the spinal cord [19]. Thus, it is necessary to expand the spinal canal and resect the ligamentum flavum to reduce posterior compression. However, in laminoplasty performed currently, the ligamentum flavum is preserved.

For CSM patients with HLF or OLF, our aim was complete removal of the ligamentum flavum and reconstruction of the bony laminar arch with rigid fixation; thus, we developed the new posterior hybrid decompression method, in which laminoplasty was interrupted by laminectomy and fixed with a spinous process autograft employing the Centerpiece plate (Figs. 3; 4A, B; 5). As demonstrated by the significantly increased JOA score, this approach yielded a satisfactory clinical outcome, with no neurological deterioration observed in our patients. Furthermore, the JOA score recovery rate was 65.3%. The mean increase of 6.7 mm in sagittal diameter at the reconstructed levels and 3.3-mm drift-back of the spinal cord, together with the significantly increased cross-sectional area of the dual sac, demonstrated satisfactory and stable spinal cord decompression.

Taking the sagittal bowstring effect [20] into consideration and to achieve a satisfactory dural sac expansion, in our department, we perform a five-level hybrid decompression for all patients with CSM resulting from anterior plus dorsal spinal cord compression or dorsal compression only, to achieve a relatively complete decompression effect. We do not perform this hybrid decompression procedure for patients with cervical ruptured disc herniation, cervical kyphosis, cervical instability, or comorbidities such as severe diabetes mellitus, which we consider contraindications for this procedure. Considering the possibility of autograft lamina nonunion, hybrid decompression with autograft employing a Centerpiece is not indicated for patients with severe diabetes, who have low bone-regeneration ability [21] and in whom there is a high incidence of deep wound infection. In our department, we consider patients with cervical instability to be indicated for the laminectomy-plus-fusion procedure [5], [22], [23] and patients with cervical ruptured disc herniation or kyphosis to be indicated for an anterior procedure or anterior-plus-posterior approach [24], [25].

In previous studies, the most common reason for failure of laminoplasty has been restenosis owing to hinge closure [26]. With suture fixation, restenosis from premature hinge closure has been reported at rates ranging from 1.5 to 34% [12], [27]–[29]. Therefore, maintaining the expansion of the spinal canal is important to successful laminoplasty [30]. In the hybrid decompression technique presented in this study, the reconstructed laminar arch was fixed by Centerpiece plates in combination with bone block (Figs. 2B, 3, 5B), which provided immediate stabilization of the laminae. During the follow-up period, there was little change in the enhanced spinal canal (Fig. 5A), indicating satisfactory clinical efficacy of the procedure. The bone block dislocation that has been described in the literature [29] was not observed, which may have been accomplished by the rigid fixation of the bone block on the Centerpiece plate by one mini-screw (Fig. 2A).

Generally, autograft is the ideal osteoconductive and osteoinductive material [31], [32]. Therefore, to facilitate early bone osteosynthesis, we placed an autologous spinous process block on the open side and spinous process chips on the hinge (Figs. 2A, B; 5B). In this study, 6 months after surgery, bone healing rates were 100% on the hinge and 92.2% on the open side. This could also be the reason why no instrumentation dislodgement was observed. On the open side, bone blocks 10 to 14 mm in length were used to produce a mean dorsal spinal cord shift of more than 3 mm and a mean sagittal canal diameter of more than 4 mm, which have been associated with good surgical outcomes [18], [33], [34].

The hybrid decompression procedure partially reestablished the posterior wall of the spinal canal. However, MRI data 1 year after surgery and data from the final follow-up records showed that all patients had a satisfactory dural sac expansion and that no notably compressed concave defects on dorsal side of dural sac were observed between laminoplasty levels (Fig. 1C, D).

Segmental motor paralysis, or so-called C5 palsy, is seen occasionally in patients treated with laminoplasty, and mean incidences of 5–8% have been reported [35]–[37]. The exact etiology of C5 palsy remains unclear; possible causes include the nerve root injury and segmental spinal cord disorder [15]. In the present series with the hybrid decompression technique, no patient developed a C5 palsy postoperatively. We hypothesize that this lower incidence of C5 palsy with the hybrid technique may be caused by resection of the ligamentum flavum.

Axial neck pain is the most frequent complaint after cervical laminoplasty, with reported rates of 6–60% [15], [38]–[40]. The exact cause of neck/shoulder pain is unclear, but the imbalance of posterior neck muscle distribution, the sinking or nonunion of the hinge of the expanded laminae, and inadequate dural expansion may be the related factors [41]–[43]. VAS scores in this study indicate that four patients (3.5%) had mild axial pain after surgery. The spinous process resection performed in this hybrid technique allows for the collateral paravertebral muscles to become symmetrically distributed become wound closure, and rigid fixation and spinous process autografts causes sinking or nonunion of the laminae to occur only rarely. The above may explain the experience of only mild axial neck pain postoperatively, but further investigation is needed.

No postoperative kyphosis was noted in our patients, which was similar to the overall rate of 0–10% that has been reported after laminoplasty [11], [33], [44]. The rate of 36.8% for loss of cervical lordosis postoperatively was within the reported range of 22–53% for laminoplasty in the literature [45]. The 8.8% decrease in cervical ROM after the hybrid decompression in this series compared favorably to that reported after laminoplasty (range, 12.0–51.0%) [33], [45]. Moreover, no patient complained of limited neck motion.

The main shortcoming of this retrospective study was the lack of a control group; thus, this hybrid decompression technique cannot be recommended over other posterior surgical options. The second weakness was the absence of an independent radiologist to review the postoperative films. Further randomized clinical trial studies from both mechanical and clinical aspects with long-term follow-up are required.

## Conclusions

The results of the current study suggest that this posterior hybrid decompression, laminoplasty interrupted by laminectomy and fixed by Centerpiece plate combined with spinous process autograft, may be a safe and effective treatment for cervical myelopathy with hypertrophy or ossification of the ligamentum flavum.

## Supporting Information

Video S1 **The surgical procedure video of this cervical hybrid decompression protocol.**
(WMV)

## References

[1] Tracy JA, Bartleson JD (2010) Cervical spondylotic myelopathy. Neurologist 16: 176–87. 20445427 10.1097/NRL.0b013e3181da3a29

[2] Toledano M, Bartleson JD (2013) Cervical spondylotic myelopathy. Neurol Clin 31: 287–305. 23186905 10.1016/j.ncl.2012.09.003

[3] Wang L, Song Y, Liu L, Liu H, Kong Q, et al (2012) Clinical outcomes of two different types of open-door laminoplasties for cervical compressive myelopathy: a prospective study. Neurol India 60: 210–6. 22626706 10.4103/0028-3886.96403

[4] Konya D, Ozgen S, Gercek A, Pamir MN (2009) Outcomes for combined anterior and posterior surgical approaches for patients with multisegmental cervical spondylotic myelopathy. J Clin Neurosci 16: 404–9. 19153044 10.1016/j.jocn.2008.07.070

[5] Muthukumar N (2012) Surgical management of cervical spondylotic myelopathy. Neurol India 60: 201–9. 22626705 10.4103/0028-3886.96402

[6] Mummaneni PV, Kaiser MG, Matz PG, Anderson PA, Groff MW, et al (2009) Cervical surgical techniques for the treatment of cervical spondylotic myelopathy. J Neurosurg Spine 11: 130–41. 19769492 10.3171/2009.3.SPINE08728

[7] Singhal U, Jain M, Jaiswal AK, Behari S (2009) Unilateral ossified ligamentum flavum in the high cervical spine causing myelopathy. Indian J Orthop 43: 305–8. 19838355 10.4103/0019-5413.49385 PMC2762168

[8] Shiraishi T (2002) Skip laminectomy–a new treatment for cervical spondylotic myelopathy, preserving bilateral muscular attachments to the spinous processes: a preliminary report. Spine J 2: 108–15. 14588269 10.1016/s1529-9430(01)00118-8

[9] Lu JJ (2007) Cervical laminectomy: technique. Neurosurgery 60 (1 Supp1): S149–53. 10.1227/01.NEU.0000249219.72956.C7 17204877

[10] O’Brien MF, Peterson D, Casey AT, Crockard HA (1996) A novel technique for laminoplasty augmentation of spinal canal area using titanium miniplate stabilization. A computerized morphometric analysis. Spine (Phila Pa 1976) 21: 474–83 discussion 484. 8658252 10.1097/00007632-199602150-00012

[11] Hirabayashi K, Watanabe K, Wakano K, Suzuki N, Satomi K, et al (1983) Expansive open-door laminoplasty for cervical spinal stenotic myelopathy. Spine (Phila Pa 1976) 8: 693–9. 6420895 10.1097/00007632-198310000-00003

[12] Rhee JM, Register B, Hamasaki T, Franklin B (2011) Plate-only open door laminoplasty maintains stable spinal canal expansion with high rates of hinge union and no plate failures. Spine (Phila Pa 1976) 36: 9–14. 21192219 10.1097/BRS.0b013e3181fea49c

[13] Iwakura M, Yamamoto K, Nagashima T, Tamaki N (1999) Surgical technique and long-term follow-up of laminoplasty using titanium miniplates. No Shinkei Geka 27: 525–31. 10396735

[14] Jiang L, Chen W, Chen Q, Xu K, Wu Q, et al (2012) Clinical application of a new plate fixation system in open-door laminoplasty. Orthopedics 35: e225–31. 22310411 10.3928/01477447-20120123-07

[15] Chen G, Luo Z, Nalajala B, Liu T, Yang H (2012) Expansive open-door laminoplasty with titanium miniplate versus sutures. Orthopedics 35: e543–8. 22495857 10.3928/01477447-20120327-24

[16] Hirabayashi K, Miyakawa J, Satomi K, Maruyama T, Wakano K (1981) Operative results and postoperative progression of ossification among patients with ossification of cervical posterior longitudinal ligament. Spine (Phila Pa 1976) 6: 354–64. 6792717 10.1097/00007632-198107000-00005

[17] Machino M, Yukawa Y, Ito K, Nakashima H, Kato F (2011) Dynamic changes in dural sac and spinal cord cross-sectional area in patients with cervical spondylotic myelopathy: cervical spine. Spine (Phila Pa 1976) 36: 399–403. 20890264 10.1097/BRS.0b013e3181d2510b

[18] Sodeyama T, Goto S, Mochizuki M, Takahashi J, Moriya H (1999) Effect of decompression enlargement laminoplasty for posterior shifting of the spinal cord. Spine (Phila Pa 1976) 24: 1527–31 discussion 1531–2. 10457571 10.1097/00007632-199908010-00005

[19] Fehlings MG, Skaf G (1998) A review of the pathophysiology of cervical spondylotic myelopathy with insights for potential novel mechanisms drawn from traumatic spinal cord injury. Spine (Phila Pa 1976) 23: 2730–7. 9879098 10.1097/00007632-199812150-00012

[20] Naderi S, Ozgen S, Pamir MN, Ozek MM, Erzen C (1998) Cervical spondylotic myelopathy: surgical results and factors affecting prognosis. Neurosurgery 43: 43–9 discussion 49–50. 9657187 10.1097/00006123-199807000-00028

[21] Sathyendra V, Darowish M (2013) Basic science of bone healing. Hand Clin 29: 473–81. 24209946 10.1016/j.hcl.2013.08.002

[22] Rhee JM, Basra S (2008) Posterior surgery for cervical myelopathy: laminectomy, laminectomy with fusion, and laminoplasty. Asian Spine J 2: 114–26. 20404967 10.4184/asj.2008.2.2.114 PMC2852088

[23] Gok B, McLoughlin GS, Sciubba DM, McGirt MJ, Chaichana KL, et al (2009) Surgical management of cervical spondylotic myelopathy with laminectomy and instrumented fusion. Neurol Res 31: 1097–101. 19215639 10.1179/174313209X383277

[24] Roberts MP, Robinson F (1996) The current treatment of cervical disc rupture. Conn Med 60: 395–8. 8758657

[25] Geck MJ, Eismont FJ (2002) Surgical options for the treatment of cervical spondylotic myelopathy. Orthop Clin North Am 33(2) 329–48. 10.1016/s0030-5898(02)00002-0 12389279

[26] Park AE, Heller JG (2004) Cervical laminoplasty: use of a novel titanium plate to maintain canal expansion–surgical technique. J Spinal Disord Tech 17: 265–71. 15280753 10.1097/01.bsd.0000095401.27687.c0

[27] Matsumoto M, Watanabe K, Tsuji T, Ishii K, Takaishi H, et al (2008) Risk factors for closure of lamina after open-door laminoplasty. J Neurosurg Spine 9: 530–7. 19035744 10.3171/SPI.2008.4.08176

[28] Satomi K, Ogawa J, Ishii Y, Hirabayashi K (2001) Short-term complications and long-term results of expansive open-door laminoplasty for cervical stenotic myelopathy. Spine J 1: 26–30. 14588365 10.1016/s1529-9430(01)00008-0

[29] Jiang JL, Li XL, Zhou XG, Lin H, Dong J (2012) Plate-only open-door laminoplasty with fusion for treatment of multilevel degenerative cervical disease. J Clin Neurosci 19: 804–9. 22475766 10.1016/j.jocn.2011.09.021

[30] Deutsch H, Mummaneni PV, Rodts GE, Haid RW (2004) Posterior cervical laminoplasty using a new plating system: technical note. J Spinal Disord Tech 17: 317–20. 15280762 10.1097/01.bsd.0000091070.73042.23

[31] Yang SC, Yu SW, Tu YK, Niu CC, Chen LH, et al (2007) Open-door laminoplasty with suture anchor fixation for cervical myelopathy in ossification of the posterior longitudinal ligament. J Spinal Disord Tech 20: 492–8. 17912125 10.1097/BSD.0b013e318033e844

[32] Yang SC, Niu CC, Chen WJ, Wu CH, Yu SW (2008) Open-door laminoplasty for multilevel cervical spondylotic myelopathy: good outcome in 12 patients using suture anchor fixation. Acta Orthop 79: 62–6. 18283574 10.1080/17453670710014770

[33] Tani S, Isoshima A, Nagashima Y, Tomohiko NR, Abe T (2002) Laminoplasty with preservation of posterior cervical elements: surgical technique. Neurosurgery 50: 97–101 discussion 101–2. 11844239 10.1097/00006123-200201000-00017

[34] Itoh T, Tsuji H (1985) Technical improvements and results of laminoplasty for compressive myelopathy in the cervical spine. Spine (Phila Pa 1976) 10: 729–36. 3909451 10.1097/00007632-198510000-00007

[35] Sakaura H, Hosono N, Mukai Y, Ishii T, Yoshikawa H (2003) C5 palsy after decompression surgery for cervical myelopathy: review of the literature. Spine (Phila Pa 1976) 28: 2447–51. 14595162 10.1097/01.BRS.0000090833.96168.3F

[36] Satomi K, Nishu Y, Kohno T, Hirabayashi K (1994) Long-term follow-up studies of open-door expansive laminoplasty for cervical stenotic myelopathy. Spine (Phila Pa 1976) 19: 507–10. 8184342 10.1097/00007632-199403000-00003

[37] Tsuji T, azuma T, suoka K, suoka H, tosuneya T, et al (2007) Retrospective cohort study between selective and standard C3–7 laminoplasty. Minimum 2-year follow-up study. Eur Spine J 16: 2072–7. 17726618 10.1007/s00586-007-0428-5 PMC2140119

[38] Kawaguchi Y, Kanamori M, Ishihara H, Ohmori K, Nakamura H, et al. (2003) Minimum 10-year follow up after en bloc cervical laminoplasty. Clin Orthop Relat Res Jun: 129–39. 10.1097/01.blo.0000069889.31220.62 12782868

[39] Ratliff JK, Cooper PR (2003) Cervical laminoplasty: a critical review. J Neurosurg 98 (3 Suppl): 230–8. 10.3171/spi.2003.98.3.0230 12691377

[40] Sun Y, Zhang F, Wang S, Zhang L, Pan S, et al (2010) Open door expansive laminoplasty and postoperative axial symptoms: a comparative study between two different procedures. Evid Based Spine Care J 1: 27–33. 22956925 10.1055/s-0030-1267065 PMC3427960

[41] Wang SJ, Jiang SD, Jiang LS, Dai LY (2011) Axial pain after posterior cervical spine surgery: a systematic review. Eur Spine J 20: 185–94. 20941514 10.1007/s00586-010-1600-x PMC3030716

[42] Yoshida M, Tamaki T, Kawakami M, Nakatani N, Ando M, et al (2002) Does reconstruction of posterior ligamentous complex with extensor musculature decrease axial symptoms after cervical laminoplasty. Spine (Phila Pa 1976) 27: 1414–8. 12131738 10.1097/00007632-200207010-00008

[43] Yukawa Y, Kato F, Ito K, Horie Y, Hida T, et al (2007) Laminoplasty and skip laminectomy for cervical compressive myelopathy: range of motion, postoperative neck pain, and surgical outcomes in a randomized prospective study. Spine (Phila Pa 1976) 32: 1980–5. 17700444 10.1097/BRS.0b013e318133fbce

[44] Matsunaga S, Sakou T, Nakanisi K (1999) Analysis of the cervical spine alignment following laminoplasty and laminectomy. Spinal Cord 37: 20–4. 10025690 10.1038/sj.sc.3100749

[45] Steinmetz MP, Resnick DK (2006) Cervical laminoplasty. Spine J 6 (6 Suppl): 274S–281S. 10.1016/j.spinee.2006.04.023 17097547

